# Splenic Infarction Due to Epstein-Barr Virus: A Case Report and Literature Review

**DOI:** 10.7759/cureus.58414

**Published:** 2024-04-16

**Authors:** Adit Singhal, Kelly I Suchman, Aaron Rhee, Himanshu Patel, Awais Paracha, Vedika Agrawal, Jessica Cohen

**Affiliations:** 1 Internal Medicine, Northwell Health, New Hyde Park, USA; 2 Gastroenterology, Northwell Health, New Hyde Park, USA; 3 Internal Medicine, Zucker School of Medicine at Hofstra/Northwell, Hempstead, USA; 4 Pediatrics, Ohio State University, Nationwide Children's Hospital, Columbus, USA

**Keywords:** ebv, case report, epstein-barr virus, splenic infarction, infectious mononucleosis

## Abstract

Splenic infarction is a rare and likely underdiagnosed complication of Epstein-Barr virus (EBV)-associated infectious mononucleosis (IM). Here, we describe an 18-year-old Guyanese male with persistent severe left-sided abdominal pain found to be EBV positive and have a large splenic infarct, along with a transient decrease in protein C, protein S, and antithrombin III activity levels. He was treated with supportive care and anticoagulated with heparin and apixaban. We review prior reports and perspectives on underlying pathophysiology, diagnosis, and the management of these cases, which likely do not require anticoagulation but may be considered on a per-case basis.

## Introduction

Infectious mononucleosis (IM), caused by Epstein-Barr virus (EBV), is characterized by fever, sore throat, lymphadenopathy, and fatigue. While most cases of IM are self-limiting and resolve without complication, rare complications such as splenic rupture or infarction have been described in 0.1-0.5% of cases [1,2]. EBV-associated splenic infarction has been reported in 29 cases per our literature review, yet the mechanism by which it occurs remains unclear. Although management typically involves supportive care, infarction should be considered in patients presenting with IM and abdominal pain to prevent potential complications such as rupture, abscess, and sepsis [3,4]. Here, we present an EBV-positive young adult male with abdominal pain, found to have a splenic infarct along with a transient decrease in protein C, S, and antithrombin III (ATIII) activity levels. 

This article was previously posted to the Authorea preprint server on March 25, 2023. 

## Case presentation

An 18-year-old male with no past medical history presented in the emergency department (ED) with persistent left-sided abdominal pain for eight days with associated subjective fevers, nausea, and non-bilious, non-bloody emesis. His pain was described as burning and stabbing diffusely and localized more to the left upper quadrant (LUQ). He denied any recent illness, diarrhea, cough, recent travel, or other symptoms. His pain was well controlled with oral acetaminophen. The patient was in college, learning remotely, and denied recent sick contacts. He took no medications, had no family history of autoimmune disorders, and denied alcohol, tobacco, or drug use. 

**Figure 1:**
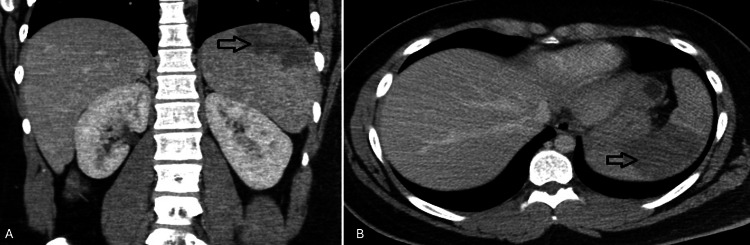
CT scan of the abdomen demonstrating splenic infarction. (A) Coronal view and (B) transverse view.

On presentation, he was afebrile (37.8°C) and tachycardic to 118 beats per minute. The exam was notable for LUQ and right lower quadrant abdominal tenderness. No lymphadenopathy, tonsillar edema/exudates, petechial hemorrhages, splenomegaly, or hepatomegaly were noted. Computed tomography (CT) revealed a mildly enlarged spleen measuring 13.8 cm with a large wedge-shaped region within the superior aspect of the spleen, compatible with infarct (Figures 1A, 1B). 

Serology confirmed IM due to acute EBV infection with an elevated EBV immunoglobulin M (IgM) at >160.0 U/mL. Human immunodeficiency virus (HIV), coronavirus disease 2019 (COVID-19), and cytomegalovirus (CMV) tests were negative. Mild elevations in total white cell count (11.09 K/uL), prothrombin time (16.3 seconds), activated partial thromboplastin time (27.2 seconds), and international normalized ratio (1.38) were noted with unremarkable electrolyte panel. Liver function tests showed mild transaminitis with aspartate aminotransferase (AST) (51 U/L) and alanine transaminase (ALT) (74 U/L), slight elevation in lactate dehydrogenase (LDH) to 480 U/L, but normal haptoglobin and total and indirect bilirubin levels. Anti-nuclear factor titers were elevated (1:680) with speckling but with negative lupus anticoagulant (1.09, normal values < 1.2, with dilute Russell’s viper venom method). Decreased protein C antigen and activity levels (65% and 73%, respectively), protein S free activity level (49%), and a low but normal antithrombin III (ATIII) antigen level (19 mg/dL, normal values 19-31) with decreased activity (65%) were also found. 

Unfractionated heparin was started in the ED, pain was well controlled with acetaminophen as needed, and the patient was discharged home on day 4 with apixaban 10 mg twice daily for seven days (loading dose) followed by 5 mg daily. He was evaluated in the clinic two months later during which magnetic resonance imaging (MRI) showed no change in spleen size or infarct, labs showed a still positive but reduced EBV IgM titer (56.0 U/mL). As the patient was asymptomatic following discharge, it was concluded that further follow-up was unnecessary. Consequently, apixaban was discontinued due to the lack of evidence supporting long-term anticoagulation for resolving previous infarcts. 

## Discussion

Previous cases of IM-associated splenic infarcts show several similarities; patients often present with a fever and abdominal pain (most commonly LUQ), and have splenomegaly on exam, although up to 30% of patients may be asymptomatic (Table 1) [5]. In our case, unfractionated heparin was initiated in the ED, and apixaban was started prophylactically due to a dearth of prior evidence and our patient’s decrease in protein C, S, and ATIII activity levels, with the anticipation of likely discontinuation on outpatient follow-up. Given that almost all prior reported cases improved with supportive management only, therapeutic anticoagulation is likely unnecessary in management but should still be considered on a case-by-case basis. 

**Table 1:** Literature review of splenic infarction in EBV infectious mononucleosis M*, *male; F*, *female; LUQ, left upper quadrant; RUQ, right upper quadrant; N/A, not applicable; ASA, aspirin; EBV, Epstein-Barr virus

	Year	Age/Sex	Abdominal Pain	Splenomegaly	Splenic Rupture	Splenectomy	Anticoagulation	Survival
Chevat et al. [6]	1961	N/A	LUQ	Yes	Yes	Yes	N/A	Yes
Guibaud et al. [7]	1983	34/M	LUQ	N/A	N/A	Yes	N/A	Yes
Boivin and Bernard [8]	1990	19/F	LUQ	Yes	No	Yes	N/A	Yes
Garten et al. [9]	1992	57/F	No	Yes	No	Yes	N/A	Yes
Trevenzoli et al. [10]	2001	17/M	LUQ	Yes	No	No	No	Yes
Symeonidis et al. [11]	2001	17/M	LUQ	Yes	No	No	No	Yes
Kim and Kopelman [12]	2005	40/M	LUQ	No	No	No	No	Yes
van Hal et al. [13]	2005	35/F	LUQ	No	No	No	No	Yes
Benz et al. [14]	2007	19/F	LUQ	Yes	No	No	No	Yes
Hunt et al. [4]	2010	29/F	LUQ	Yes	No	No	No	Yes
Breuer et al. [15]	2010	13/M	LUQ	Yes	No	No	No	Yes
Cull and Stein [16]	2012	18/F	LUQ	Yes	Yes	No	No	Yes
Gang et al. [17]	2013	7/F	RUQ	Yes	No	No	No	Yes
Gavriilaki et al. [18]	2013	17/M	LUQ	Yes	No	No	No	Yes
Kobe et al. [19]	2013	22/M	RUQ	Yes	No	No	No	Yes
Mackenzie and Liebmann [20]	2013	18/M	LUQ	Yes	No	No	No	Yes
Li et al. [21]	2014	19/F	Upper	Yes	No	No	No	Yes
Bhattarai et al. [22]	2014	16/M	Epigastric	Yes	No	No	No	Yes
Machado et al. [23]	2015	24/M	LUQ	Yes	No	No	No	Yes
Heo et al. [24]	2016	20/F	LUQ	No	No	No	No	Yes
Naviglio et al. [25]	2016	14/M	LUQ	Yes	No	No	No	Yes
Noor et al. [26]	2017	25/F	LUQ	Yes	No	No	No	Yes
Li et al. [27]	2018	24/F	LUQ	No	No	No	No	Yes
		20/M	LUQ	No	No	No	No	Yes
		25/F	LUQ	Yes	No	No	No	Yes
Pervez et al. [28]	2020	20/M	LUQ	No	No	No	No	Yes
Turrian [29]	2021	24/M	LUQ	N/A	No	No	7 Days	Yes
Nishioka et al. [30]	2022	19/M	LUQ	No	No	No	No	Yes
Sowka and Mali [31]	2022	60/M	Left Side	N/A	No	No	Lovenox to ASA	Yes

The mechanism behind splenic infarction with EBV infection remains unclear. Splenic histopathology results are limited but have noted splenic lymphoid hyperplasia, partial fibrosis, and sinus congestion without thrombus formation. This hypercellularity has been thought to disrupt splenic sinus infrastructure, causing shifts in blood flow that are unable to meet the increased oxygen demand of an enlarged spleen and thereby increase the risk of ischemia [6,9,32,33]. Earlier case reports and studies have also suggested that transient coagulopathic states may be involved, with instances of decreased activity of proteins C and S, positive lupus anticoagulant and anticardiolipin antibodies, positive antiphospholipid antibodies, and increased factor VIII [4,15-17,23,27]. However, thrombophilia studies in most reports were unremarkable. An underlying predisposition in conjunction with the above may predispose certain patients to be more coagulopathic including hereditary spherocytosis, sickle cell trait, pyruvate kinase deficiency, or co-infection with other viruses [8,21,32,33]. However, most reports showed unremarkable thrombophilia studies and no signs of thrombus formation in other organ systems. Furthermore, almost all cases exhibited splenomegaly. Thus, we suspect architectural changes with lymphoproliferation within splenic sinuses likely contribute to infarction more than a transient thrombogenic state. 

Point-of-care ultrasound is becoming more utilized as a first-line imaging modality in the acute setting for abdominal pain [7], but given that a 2009 retrospective study showed ultrasound sensitivity to be only 18% [2], contrast-enhanced CT should be the test of choice. MRI may be considered for pediatric populations in particular if ionizing radiation is a concern. Few prior studies reported follow-up imaging like ours, but results vary from improvement to no significant changes. Repeat CT or MRI is likely an unnecessary and costly test with little benefit in guiding further management. Patients should avoid contact sports to reduce the risk of splenic rupture for a minimum of three weeks after onset of clinical symptoms [34], although splenomegaly has been reported in cases to persist for longer (>8 weeks in our case). Clinicians may use ultrasound to help gauge improvement in splenomegaly if a patient plans to return to contact sports. 

## Conclusions

Although rare, splenic infarctions associated with IM should be considered in patients presenting with abdominal pain. There are currently no guidelines for diagnosis and management of these patients. Despite some cases having a self-limiting transient thrombogenic phase which doesn’t require anticoagulation, clinicians should be aware of potential complications including abscess and rupture. 
